# An updated meta-analysis of head-to-head trials comparing the efficacy, safety, and adherence of mirabegron and vibegron in overactive bladder

**DOI:** 10.1097/MD.0000000000046109

**Published:** 2026-05-12

**Authors:** Wei-Zhen Bai, Shi-Yu Zhou, Yuan-Yuan Mou, Liao Peng, Ling-Li Li

**Affiliations:** a West China School of Nursing, Sichuan University/Department of Anesthesiology, West China Hospital, Sichuan University, Chengdu, China; b Department of Nursing, West China Hospital, Sichuan University/West China School of Nursing, Sichuan University, Chengdu, China; c Department of Urology, West China Hospital, Sichuan University, Chengdu, China.

**Keywords:** mirabegron, overactive bladder, vibegron, β3-adrenergic agonist

## Abstract

**Background:**

This updated meta-analysis aimed to compare the efficacy, safety, and treatment adherence of mirabegron and vibegron in patients with overactive bladder with head-to-head trials.

**Methods:**

A systematic literature search was conducted in PubMed, Embase, ClinicalTrials.gov, and the Cochrane Library from January 1, 2016 to July 6, 2025. Comparative studies evaluating mirabegron versus vibegron were included. Study selection followed the PICOS framework, and quality assessment was performed using the Cochrane Handbook for randomized controlled trials (RCTs) and the Newcastle–Ottawa Scale for non-RCTs. Outcomes included changes in voiding diary parameters, urodynamic measures, overactive bladder symptom scores, adverse events, and treatment adherence. Statistical analyses were conducted using Review Manager 5.3.

**Results:**

Six studies met the inclusion criteria. Vibegron was associated with a greater reduction in daily urgency episodes (standardized mean difference [SMD] = 0.37, *P* = .0006) and urinary urge incontinence (UUI) episodes (SMD = 0.33, *P* = .006) compared to mirabegron. However, no significant differences were found in other efficacy parameters or overall safety profiles. Vibegron demonstrated better adherence, with a higher continuation rate and lower discontinuation rate.

**Conclusion:**

Recent data indicated that vibegron demonstrated superior efficacy in reducing urgency and UUI episodes while maintaining a safety profile comparable to mirabegron. Furthermore, vibegron is associated with improved treatment adherence. However, additional RCTs are necessary to confirm these findings.

## 1. Introduction

Overactive bladder (OAB) is a prevalent condition characterized by urinary urgency, increased frequency, nocturia, and, in many cases, urge incontinence.^[1]^ It significantly affects patients’ quality of life, leading to social embarrassment, psychological distress, and reduced daily functioning.^[2]^ Pharmacological management primarily involves β3-adrenergic receptor agonists, including mirabegron^[3]^ and vibegron,^[4]^ which have emerged as effective alternatives to antimuscarinic agents due to their favorable efficacy and safety profiles.

Mirabegron, the first β3-adrenergic agonist approved for OAB treatment, has demonstrated efficacy in improving urinary symptoms with a lower incidence of anticholinergic side effects.^[5]^ Vibegron, a newer agent in the same class, offers potential advantages, including a faster onset of action and improved tolerability.^[6]^ However, direct comparative data assessing the relative efficacy, safety, and adherence between these 2 agents remain limited.

Previous network meta-analyses^[7,8]^ have attempted to compare the 2 drugs; however, these analyses relied on indirect comparisons, drawing conclusions based on separate placebo-controlled trials rather than direct head-to-head studies. To address this issue, 2 previous reviews^[9,10]^ have evaluated the safety and efficacy of mirabegron and vibegron for the OAB with the head-to-head trials (with only 3 studies), but several new head-to-head studies^[11–13]^ have since been published, and their conclusions vary to some extent. Therefore, with the increasing availability of direct comparative trials published in recent years,^[11–16]^ a reassessment of their relative efficacy and safety is warranted.

The updated meta-analysis aims to comprehensively compare mirabegron and vibegron in terms of symptom improvement, adverse event profiles, and treatment adherence based on data from head-to-head clinical studies. The findings may provide valuable insights for clinicians in selecting the most appropriate therapy for OAB management.

## 2. Materials and methods

### 2.1. Study selection

**Figure 1.**
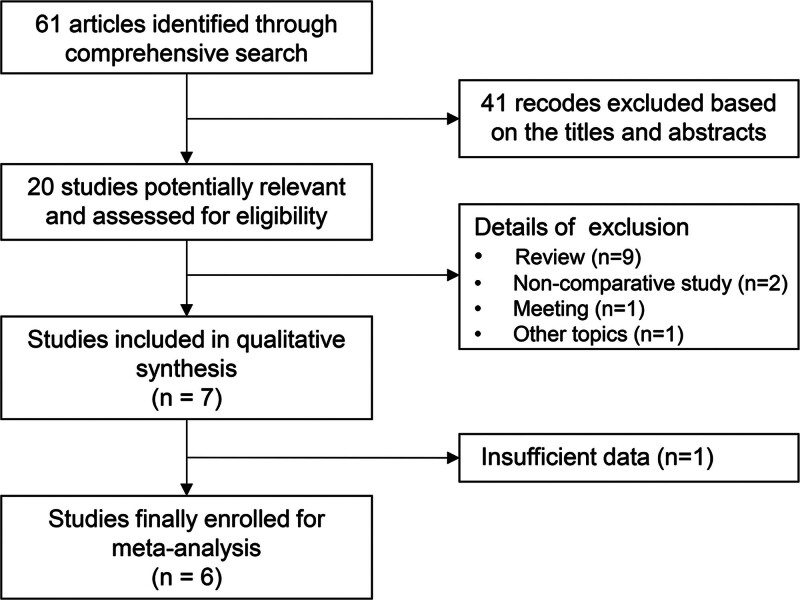
PRISMA flowchart of study selection.

Ethical approval was not required for this study, as all included studies were publicly accessible. The study was conducted in accordance with PRISMA guidelines^[17]^ (Fig. 1). A comprehensive search was performed across multiple databases, including PubMed, Embase, ClinicalTrials.gov, and the Cochrane Library Central Register of Controlled Trials, covering the period from January 1, 2016 to July 6, 2025. The search strategy incorporated the following terms: (mirabegron OR vibegron OR adrenergic beta-3 receptor agonists) AND (overactive bladder OR OAB OR urinary bladder, overactive). Additionally, reference lists of eligible studies, relevant reviews, and guidelines were manually screened. Only comparative trials evaluating mirabegron versus vibegron were considered, with no restrictions on publication status or language.

### 2.2. Inclusion criteria and data extraction

The study selection process was conducted in accordance with the PICOS framework.^[18]^ However, studies such as case reports, study protocols, commentaries, or those with insufficient data were excluded. The quality of randomized controlled trial (RCT) was assessed following the Cochrane Handbook guidelines,^[19]^ while non-RCTs were evaluated using the Newcastle–Ottawa Scale.^[20]^ After finalizing the studies for meta-analysis, relevant baseline characteristics, treatment regimens, symptom score changes, and adverse events were systematically collected for further analysis. Data extraction was performed independently by 2 researchers, and their results were cross-verified. Any discrepancies were resolved through team discussions to achieve consensus.

### 2.3. Outcomes

Efficacy was assessed based on changes in voiding diary parameters, urodynamic test results, and OAB symptom scores.^[21,22]^ Safety was evaluated by analyzing reported adverse events from the included studies, such as dry mouth, constipation, and cardiovascular complications. Additionally, the continuation and discontinuation rates of the 2 treatments were calculated to further assess treatment adherence and tolerability.

### 2.4. Data analysis

All statistical analyses were conducted using Review Manager 5.3 (Cochrane Collaboration, Oxford, UK). For dichotomous outcomes, odds ratios (ORs) with 95% confidence intervals (CIs) were calculated, while standardized mean differences (SMDs) with 95% CIs were used for continuous variables. Heterogeneity among studies was assessed using the *I*^2^ statistic. A fixed-effects model was applied when heterogeneity was low (*I*^2^ < 50%), whereas a random-effects model was used for substantial heterogeneity (*I*^2^ ≥ 50%). A *P*-value of less than .05 was considered statistically significant.

## 3. Results

As illustrated in Figure 1, a total of 6 studies were included in the analysis, comprising 3 RCTs, 2 retrospective studies, and 1 cross-sectional study. The key characteristics of the selected studies are summarized in Table 1. Based on quality assessments, all included studies were deemed to be of high methodological quality (Figure S1 and Table S1, Supplemental Digital Content, https://links.lww.com/MD/Q740).

**Table 1:** The characteristics of included studies.

Author	Study design	Group (n)	Women (%)	Age (years)	Dosage	Follow-up
Mukai et al (2025)	R	Mirabegron (n = 66)	39.4	67 (26–94)	Adjusted by physician	3 years
		Vibegron (n = 66)	37.9	66.5 (23–88)	Adjusted by physician	3 years
Wada et al (2024)	RCT	Mirabegron (n = 33)	100	71.9 ± 8.8	50 mg once daily	8 weeks
		Vibegron (n = 34)	100	72.5 ± 6.4	50 mg once daily	8 weeks
Kinjo et al (2023)	RCT	Mirabegron (n = 97)	100	71.5 ± 12.6	50 mg once daily	12 weeks
		Vibegron (n = 102)	100	71.1 ± 10.8	50 mg once daily	12 weeks
Sato et al (2023)	RCT	Mirabegron (n = 45)	100	74 ± 9.2	50 mg once daily	12 weeks
		Vibegron (n = 44)	100	73 ± 8.2	50 mg once daily	12 weeks
Mukai et al (2021)	R	Mirabegron (n = 103)	46.6	71 (22–94)	Adjusted by physician	1 year
		Vibegron (n = 103)	44.7	68 (18–90)	Adjusted by physician	1 year
Chastek et al (2024)	Cross-sectional	Mirabegron (n = 2565)	69.2	74.7 ± 10.4	Adjusted by physician	238.1 ± 127.4 days
		Vibegron (n = 1711)	68.7	74.6 ± 10.3	Adjusted by physician	236.8 ± 127.8 days

R = retrospective, RCT = randomized controlled trials.

### 3.1. Efficacy

**Figure 2.**
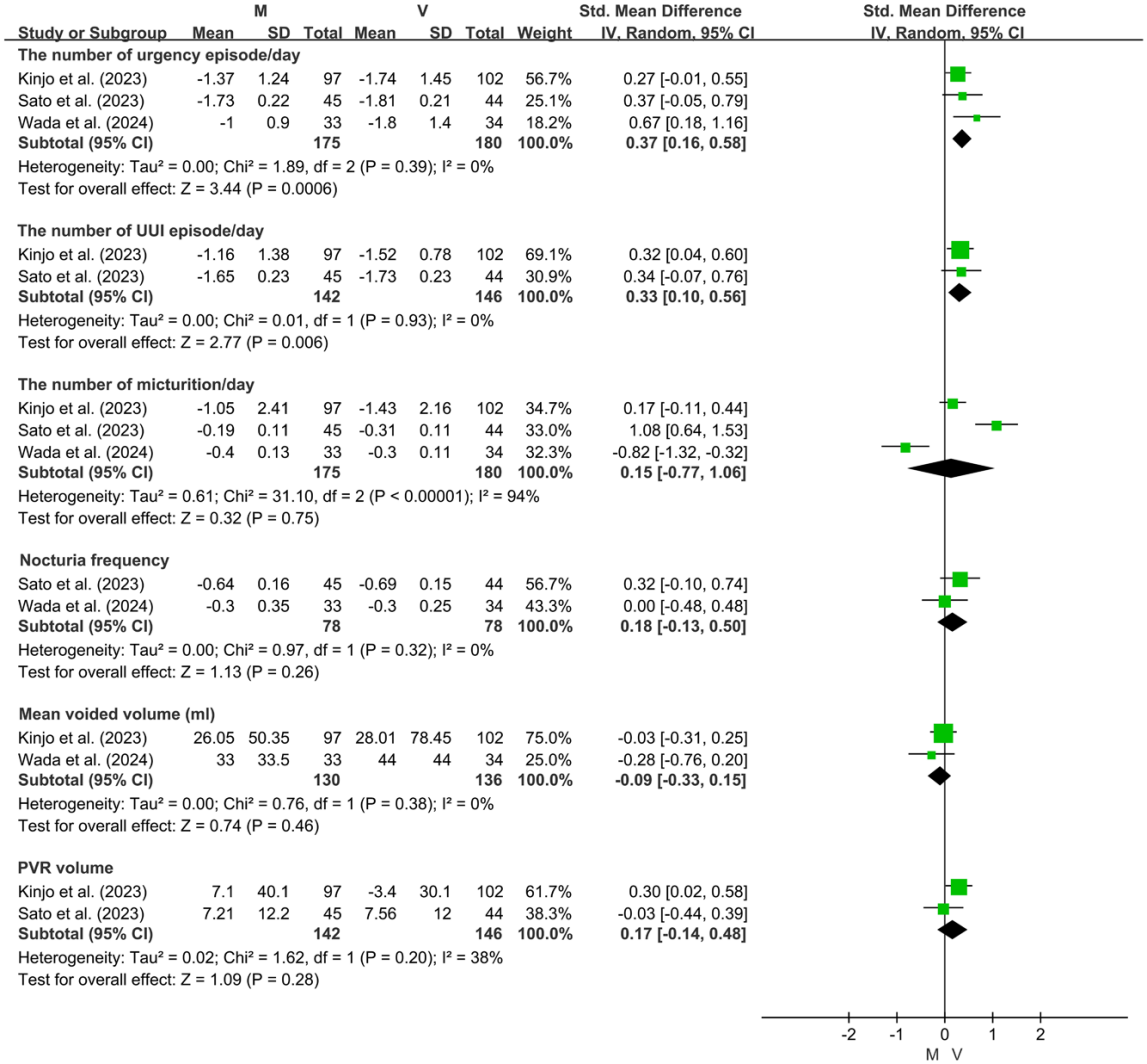
Forest plot for efficacy. M = mirabegron, PVR = post-void residual, UUI = urinary urge incontinence, V = vibegron.

**Figure 3.**
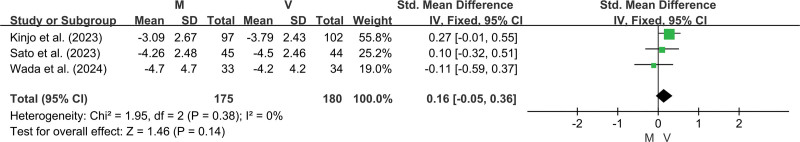
Forest plot for OAB symptom scores. OAB = overactive bladder.

Patients with OAB treated with vibegron demonstrated a greater reduction in daily urgency episodes (SMD = 0.37, 95% CI: 0.16–0.58, *P* = .0006) and urinary urge incontinence (UUI) episodes per day (SMD = 0.33, 95% CI: 0.10–0.56, *P* = .006) compared to those receiving mirabegron. However, other parameters, including the number of voids per day (*P* = .75), nocturia frequency (*P* = .26), mean voided volume (*P* = .46), and post-void residual (PVR) volume (*P* = .28), did not differ significantly between the 2 groups (Fig. 2). Additionally, no significant difference was observed in OAB symptom scores changes between the 2 treatments (*P* = .14) (Fig. 3).

### 3.2. Safety profile

**Figure 4.**
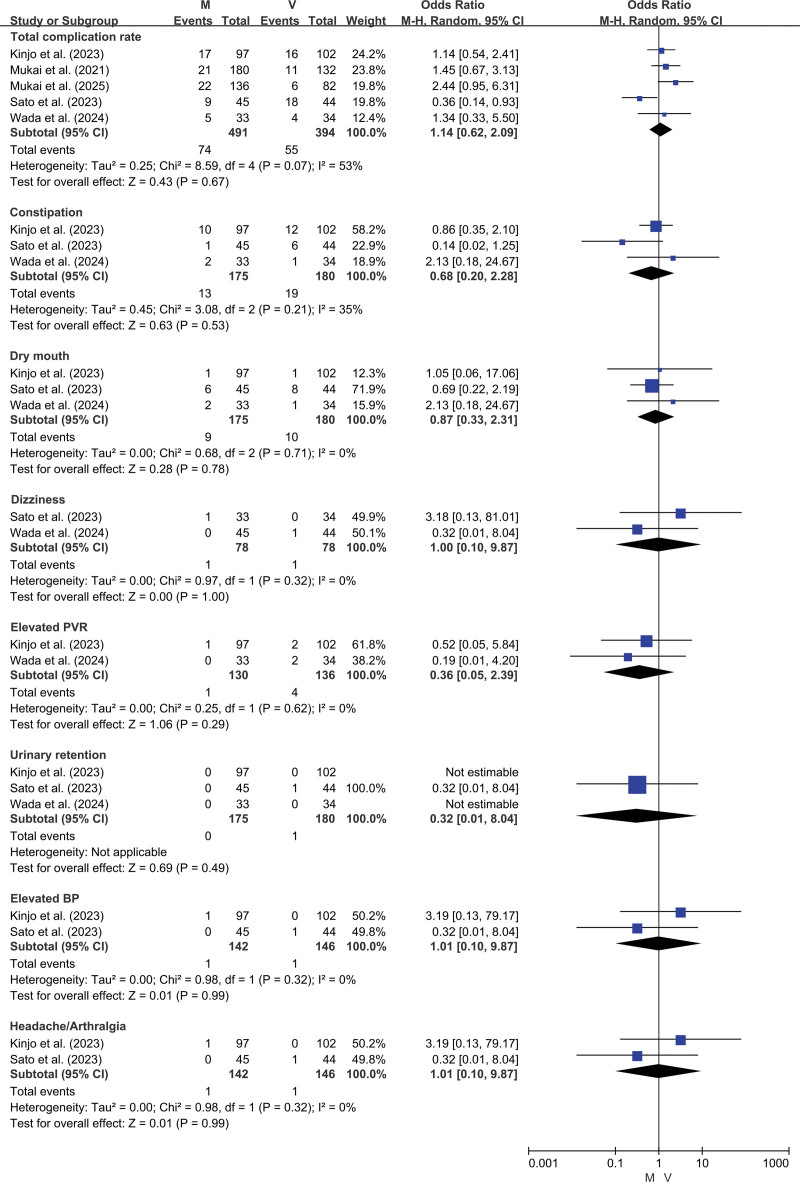
Forest plot for complications.

The overall incidence of adverse events was comparable between the mirabegron and vibegron groups (15.1% vs 13.9%, *P* = .67). As anticipated, no significant differences were found between the 2 groups in the rates of constipation, dry mouth, dizziness, elevated PVR, urinary retention, increased blood pressure, or pain (Fig. 4).

### 3.3. Treatment adherence and tolerability

**Figure 5.**
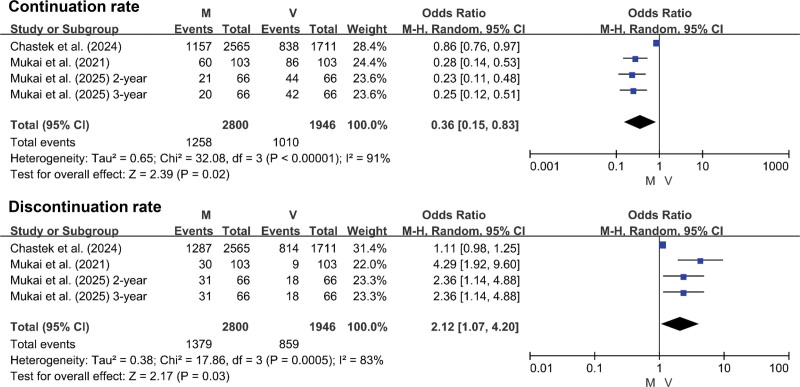
Forest plot for treatment adherence.

Patients receiving mirabegron exhibited a significantly lower continuation rate (OR = 0.36, 95% CI: 0.15–0.83, *P* = .02) and a higher discontinuation rate (OR = 2.12, 95% CI: 1.07–4.20, *P* = .03), indicating worse adherence and tolerability compared to vibegron (Fig. 5).

## 4. Discussion

The results of this meta-analysis provide a valuable contribution to the ongoing comparison of mirabegron and vibegron in the treatment of OAB. While network meta-analyses by Kennelly et al^[8]^ and He et al^[7]^ have reviewed the efficacy and safety of these 2 β3-adrenergic receptor agonists, they are limited by their reliance on indirect comparisons, drawing conclusions based on placebo-controlled trials rather than head-to-head data. Although 2 recent reviews^[9,10]^ have evaluated the comparative safety and efficacy of mirabegron and vibegron using head-to-head studies, the consensus remains unclear. Fortunately, more direct comparative trials have since been published. Therefore, there is a pressing need to update the analysis incorporating these newly available data.

The findings suggest that vibegron outperforms mirabegron in terms of reducing daily urgency and UUI episodes. Despite this, no significant differences were found between the 2 drugs regarding other efficacy measures, such as mean voided volume or PVR, and their overall safety profiles were comparable. One of the notable findings from this analysis is the better treatment adherence associated with vibegron, as evidenced by a higher continuation rate and lower discontinuation rate compared to mirabegron. This is a crucial consideration, as patient adherence to treatment regimens is often a limiting factor in the management of chronic conditions such as OAB. The reasons behind this improved adherence remain speculative but may be linked to the pharmacokinetic and pharmacodynamic properties of vibegron. In particular, vibegron has been shown to be more potent than mirabegron in stimulating cyclic adenosine monophosphate formation in cellular assays,^[23]^ with a higher maximum β3 response.^[24]^ This difference could account for its superior clinical effects, leading to better symptom control and, potentially, greater patient satisfaction and compliance. In contrast to mirabegron, vibegron does not inhibit cytochrome P450 (CYP) enzymes, an important pharmacological distinction.^[25]^ Mirabegron’s potential to inhibit CYP activity necessitates careful consideration of drug-drug interactions, particularly in patients taking other medications metabolized by these enzymes.^[25]^ This lack of CYP inhibition in vibegron may be an advantage in terms of its safety profile, especially for polypharmacy patients, which are common in OAB treatment.

The cardiovascular safety of β3-agonists is another important aspect that deserves attention.^[26]^ Both mirabegron and vibegron have been linked to cardiovascular side effects, such as palpitations and elevated blood pressure, although these AEs were generally reported to be comparable between the 2 agents.^[14]^ Interestingly, vibegron has shown to have near-exclusive β3 receptor activity, while mirabegron also exhibits modest β1 and β2 activity.^[27]^ This β1 and β2 activity in mirabegron may contribute to the observed cardiovascular AEs, such as tachycardia and elevated blood pressure.^[28]^ Vibegron’s higher selectivity for β3 receptors could make it a safer option for patients with a history of cardiovascular disease, particularly those with hypertension or arrhythmias, as its lack of β1 and β2 activity appears to mitigate the risk of cardiovascular events.^[29]^ Despite these promising findings, we agree with Kinjo et al^[15]^ and Sato et al^[14]^ that the cardiovascular safety of both agents should be monitored closely, particularly in long-term use, given the chronic nature of OAB and the need for prolonged treatment. Additionally, economic evaluations have indicated that vibegron is a cost-effective alternative to mirabegron. Specifically, vibegron was found to be cost-effective compared to mirabegron 50 mg in 98.6% and 100% of cases for commercial and Medicare payors, respectively, at a willingness-to-pay threshold of $50,000.^[30,31]^ However, future studies are needed to shed more lights on this topic.

Taken together, vibegron not only demonstrates superior efficacy in reducing urgency and UUI episodes but also appears to be more cost-effective than mirabegron. Its improved adherence and lack of CYP inhibition may contribute to better long-term treatment persistence, potentially reducing healthcare costs associated with treatment discontinuation or additional interventions. Given these advantages, vibegron could be a valuable addition to health plans for OAB management, offering both clinical and economic benefits.

Despite the strengths of this meta-analysis, several limitations should be acknowledged. First, not all of the included studies were RCTs, which may introduce bias and reduce the overall strength of the evidence. Additionally, the studies had varying follow-up durations, which may affect the long-term applicability of the findings. Another significant limitation is the lack of diverse patient populations in the included studies. Many of the trials were conducted in specific geographic regions (Japan), which may limit the generalizability of the findings to broader patient groups.

## 5. Conclusions

Vibegron demonstrated greater efficacy in reducing urgency and UUI episodes compared to mirabegron while exhibiting a similar safety profile. Furthermore, vibegron was associated with better treatment adherence. Further large-scale RCTs are needed to confirm these findings and provide more robust evidence for clinical decision-making.

## Author contributions

**Conceptualization**: Wei-Zhen Bai, Ling-Li Li.

**Data curation**: Wei-Zhen Bai, Yuan-Yuan Mou.

**Investigation**: Wei-Zhen Bai, Yuan-yuan Mou.

**Methodology**: Wei-Zhen Bai, Shi-Yu Zhou.

**Software**: Wei-Zhen Bai, Shi-Yu Zhou.

**Visualization**: Shi-Yu Zhou.

**Writing – original draft**: Wei-Zhen Bai, Liao Peng.

**Writing – review & editing**: Wei-Zhen Bai, Liao Peng, Ling-Li Li.

## Supplementary Material
