# Supplementary material for: An updated meta-analysis of head-to-head trials comparing the efficacy, safety, and adherence of mirabegron and vibegron in overactive bladder
Source: Medicine (Baltimore). 2026 May 12;104(49):e46109. doi: 10.1097/MD.0000000000046109 (PMC12688893; doi:10.1097/MD.0000000000046109)
Supplement: Supplementary file 1 [file medi-104-e46109-s001.docx]

**Supplementary Figure 1** Risk of bias of randomized controlled trials.


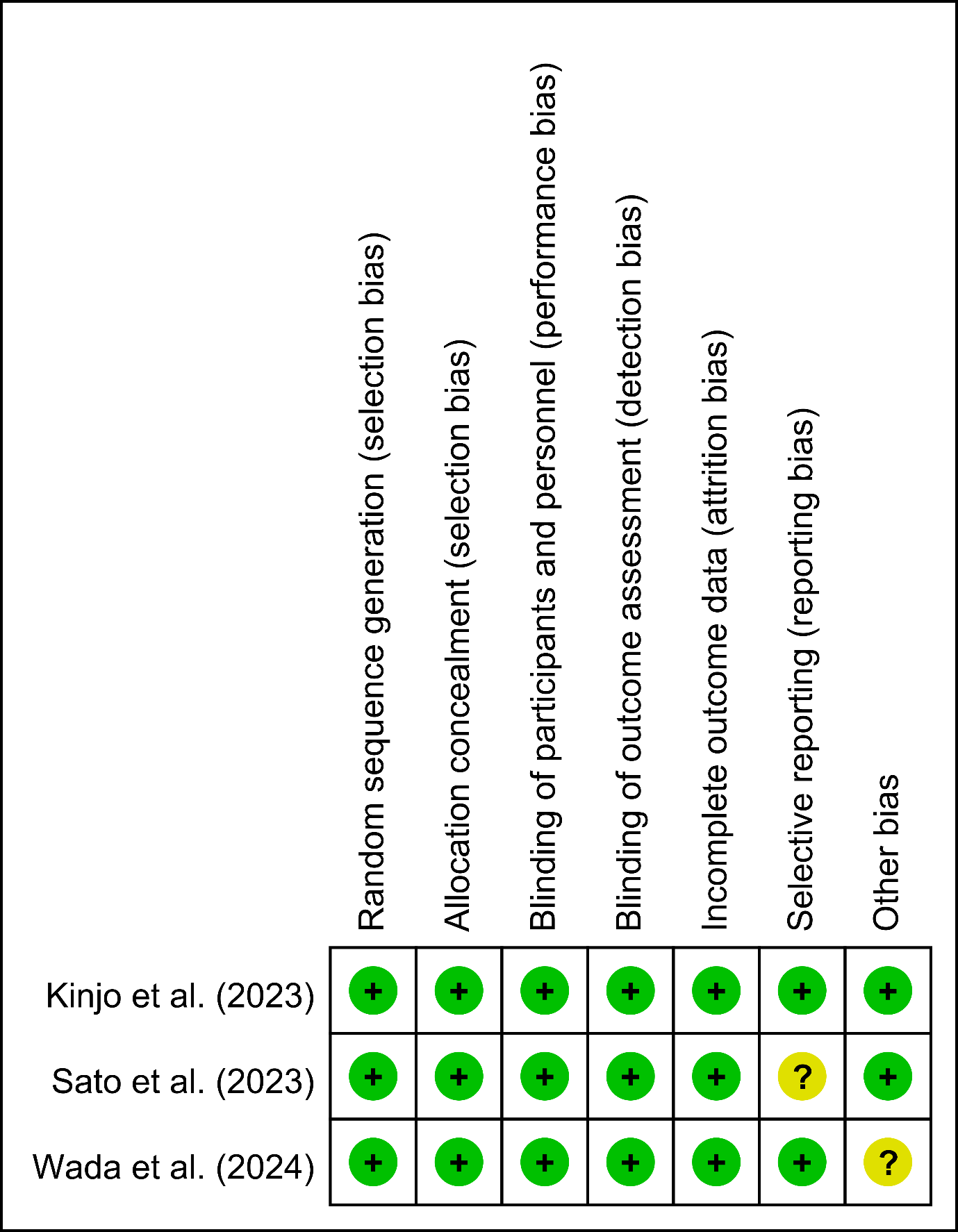


| **Supplementary Table 1** Quality assessment for non-RCTs. | | | | | | | | | | | |
| --- | --- | --- | --- | --- | --- | --- | --- | --- | --- | --- | --- |
| **NOS** | **Selection** | | | | **Comparability** | | **Exposure** | | |  | |
|  | **REC** | **SNEC** | **AE** | **DO** | **SC** | **AF** | **AO** | **FU** | **AFU** | **Results** |  |
| Mukai et al. (2025) | 1 | 1 | 1 | 1 | 1 | 1 | 1 | 0 | 1 | 8/High |  |
| Mukai et al. (2021) | 1 | 1 | 1 | 1 | 0 | 1 | 1 | 1 | 0 | 7/High |  |
| Chastek et al. (2024) | 1 | 1 | 1 | 1 | 1 | 1 | 1 | 1 | 1 | 9/High |  |
| The quality score ≥ 7 points was ranked as high.  **REC** representativeness of the cohort, **SNEC** selection of the none posed cohort, **AE** ascertainment of exposure, **DO** demonstration that outcome of interest was not present at start of study, **SC** study controls most important factors, **AF** study controls for other important factors, **AO** assessment of outcome, **FU** follow-up long enough for outcomes to occur (‘long enough’ is defined as 1 year), **AFU** adequacy of follow-up of cohort (≥ 80%). | | | | | | | | | | | |
